# Origin-of-transfer sequences facilitate mobilisation of non-conjugative antimicrobial-resistance plasmids in *Staphylococcus aureus*

**DOI:** 10.1093/nar/gkv755

**Published:** 2015-08-03

**Authors:** Frances G. O'Brien, Karina Yui Eto, Riley J. T. Murphy, Heather M. Fairhurst, Geoffrey W. Coombs, Warren B. Grubb, Joshua P. Ramsay

**Affiliations:** 1 CHIRI Biosciences Precinct, School of Biomedical Sciences, Faculty of Health Sciences, Curtin University, Bentley WA 6102, Australia; 2 Australian Collaborating Centre for *Enterococcus* and *Staphylococcus* Species (*ACCESS*) Typing and Research, School of Veterinary Sciences and Life Sciences, Murdoch University and School of Biomedical Sciences, Curtin University, Perth, Western Australia, Australia; 3 PathWest Laboratory Medicine, Fiona Stanley Hospital, Perth, Western Australia, Australia

## Abstract

*Staphylococcus aureus* is a common cause of hospital, community and livestock-associated infections and is increasingly resistant to multiple antimicrobials. A significant proportion of antimicrobial-resistance genes are plasmid-borne, but only a minority of *S. aureus* plasmids encode proteins required for conjugative transfer or Mob relaxase proteins required for mobilisation. The pWBG749 family of *S. aureus* conjugative plasmids can facilitate the horizontal transfer of diverse antimicrobial-resistance plasmids that lack Mob genes. Here we reveal that these mobilisable plasmids carry copies of the pWBG749 origin-of-transfer (*oriT*) sequence and that these *****oriT***** sequences facilitate mobilisation by pWBG749. Sequences resembling the pWBG749 *oriT* were identified on half of all sequenced *S. aureus* plasmids, including the most prevalent large antimicrobial-resistance/virulence-gene plasmids, pIB485, pMW2 and pUSA300HOUMR. *oriT* sequences formed five subfamilies with distinct inverted-repeat-2 (IR2) sequences. pWBG749-family plasmids encoding each IR2 were identified and pWBG749 mobilisation was found to be specific for plasmids carrying matching IR2 sequences. Specificity of mobilisation was conferred by a putative ribbon-helix-helix-protein gene *smpO*. Several plasmids carried 2–3 *oriT* variants and pWBG749-mediated recombination occurred between distinct *oriT* sites during mobilisation. These observations suggest this relaxase-*in trans* mechanism of mobilisation by pWBG749-family plasmids is a common mechanism of plasmid dissemination in *S. aureus*.

## INTRODUCTION

*Staphylococcus aureus* is a common cause of serious skin infections, pneumonia, sepsis and endocarditis and is carried asymptomatically by roughly a third of the human population (1,2). *S. aureus* isolates play host to a plethora of mobile genetic elements, including bacteriophage, chromosomally integrated elements, transposons and plasmids (3–6). The majority of antimicrobial-resistance genes and many of the known virulence genes are carried on plasmids (5). Through apparent acquisition of these plasmids, some strains of *S. aureus* have become resistant to almost all currently available antimicrobials, including β-lactams, macrolides, aminoglycosides and glycopeptides (7–9).

Ninety percent of *S. aureus* isolates carry at least one plasmid and ∼80% carry a plasmid >20 kb (5). Staphylococcal plasmids can be grouped by size and replication mechanism, ranging from small (<5 kb) and often cryptic rolling-circle-replication (RCR) plasmids to larger (8–50 kb) theta-replicating plasmids. Self-transmissible conjugative plasmids only represent around 5% of all sequenced *S. aureus* plasmids (10). Despite the low abundance of conjugative plasmids, phylogenomic comparisons clearly indicate that plasmid exchange between diverse *S. aureus* lineages occurs (5,11). For example, the penicillinase-encoding plasmid pMW2 has been identified in diverse, internationally isolated, community-associated methicillin-resistant *S. aureus* (CA-MRSA) lineages, including MW2, USA300, JKD6177 and JKD6159. The sequence divergence between these CA-MRSA suggests pMW2 arrived in each strain independently, despite pMW2 not carrying conjugation genes (12,13).

Conjugative DNA transfer requires the production of a mating pore between two cells and recruitment of DNA to the mating pore. The mating-pore genes usually encode a type-IV secretion system (T4SS) capable of transferring a protein-tethered DNA substrate to a recipient cell in an energy-dependent process (14). In gram-positive organisms the process of conjugation is less well understood than in gram-negative species, but several core components of the T4SS are conserved (15). Recruitment of DNA to the T4SS requires the action of a site-specific endonuclease called a relaxase or Mob protein, which makes a single-strand nick at the origin-of-transfer (*oriT*) sequence and becomes covalently attached to it (16,17). The Mob protein then recruits the DNA to the T4SS, through interaction with the T4SS coupling protein (17). Conjugative plasmids can facilitate the transfer of non-conjugative plasmids present in the same cell; a process known as mobilisation. It is generally accepted that mobilisable plasmids encode distinct Mob protein(s) that target a distinct *oriT* sequence located on the mobilisable plasmid and that this mobilisable-plasmid encoded Mob recruits the mobilisable-plasmid DNA to the mating pore (17). Documented evidence of plasmid mobilisation via this mechanism in *S. aureus* is sparse (18,19) and bioinformatics analyses have revealed that only ∼32% of *S. aureus* plasmids encode putative Mob proteins (5,11). These observations are not exclusive to *Staphylococcus*; half of all sequenced bacterial plasmids appear to lack Mob-gene loci (17,20,21). Mathematical models of plasmid persistence suggest that horizontal transfer may be essential for the long-term evolutionary maintenance of plasmids in bacterial populations, regardless of the loci they carry (22–24). Therefore the paucity of conjugation and mobilisation genes in *S. aureus* seems to conflict both with the observed distribution of plasmids in *S. aureus* and their evolutionary persistence.

Sequencing of plasmids (5) from *S. aureus* isolated from carriers of CA-MRSA in remote Western Australian communities (2) recently identified plasmids pWBG745, pWBG748 and pWBG749, which carry an uncharacterised conjugation-gene cluster, distinct from the well-characterised pSK41/pGO1 family of staphylococcal conjugative plasmids (10,25). A pWBG745-like plasmid, pBRZ01, was subsequently demonstrated to mediate conjugative transfer of vancomycin and aminoglycoside resistance in a Brazilian CA-MRSA isolate (9). We recently demonstrated that pWBG749 is capable of efficient conjugative transfer into at least seven distinct *S. aureus* lineages and is furthermore capable of mobilising several genetically distinct plasmids isolated from both community and hospital-associated *S. aureus* strains (25). None of the plasmids mobilised by pWBG749 encode recognisable Mob proteins, suggesting that these plasmids are mobilised through a distinct mechanism (25).

A non-conjugative plasmid that harbours a copy of a conjugative plasmid's *oriT* sequence can be mobilised by the *in trans* activity of the conjugative-plasmid encoded relaxase. This relaxase-*in trans* mechanism of plasmid mobilisation is routinely utilised by molecular biologists for mobilisation of plasmids using the *Escherichia coli* plasmid RP4 conjugation system (26). Nevertheless, mobilisable genetic elements that lack dedicated, *cis-*encoded Mob proteins are rarely documented in nature (27–32) and current dogma defines mobilisable plasmids as being those that carry both *oriT* and Mob gene(s) (17). In this study we define the minimal *oriT* of conjugative plasmid pWBG749 and reveal that carriage of this *oriT* by a normally non-mobilisable plasmid confers the ability to be mobilised by pWBG749. More surprisingly, we find that sequences resembling the pWBG749-family of *oriT* sequences are present on 53% of currently sequenced *S. aureus* plasmids. Our analyses suggest that *oriT* sequences have been repeatedly acquired by non-conjugative *S. aureus* plasmids during their evolution and that this relaxase-*in trans* mechanism of mobilisation is likely a dominant mechanism of horizontal plasmid transfer in *S. aureus*.

## MATERIALS AND METHODS

Strains and plasmids are listed in Supplementary Table S1. *E. coli* and *S. aureus* strains were cultured on solid or liquid LB medium, supplemented with appropriate antibiotics to maintain plasmids. Primers listed in Supplementary Table S2 were used to amplify *oriT* regions that were subsequently cloned into pLI50 as EcoRI-HindIII or EcoRI-BamHI fragments. All pLI50 constructs were sequenced using primers 21 and 22. *S. aureus* plasmid DNA was extracted as previously described (33). For construction of strains used in mobilisation experiments, pLI50 clones were electroporated into RN4220 using the method described by Schenk & Laddaga (34) and pWBG749e was subsequently introduced by conjugation and selected for by plating on media containing erythromycin (Em) and chloramphenicol (Cm) to select against donors. All conjugation assays were carried out using a modified version of the polyethylene glycol (PEG) method, which promotes cell-cell contact in liquid cultures and produces conjugation frequencies similar to filter-matings (35). Briefly, 0.5 ml of overnight donor and recipient cultures were mixed and pelleted by centrifugation at 10,000 g. Pellets were then suspended in 100 μl LB and then mixed with a 50% mixture of autoclaved BHIB and PEG 6000 and placed in a 30 ml bottle and incubated overnight at 37°C with shaking at 200 rpm. Cells were recovered by centrifugation and suspended in 1 ml LB before further dilution and plating on selective media. WBG4515 was used as a recipient and streptomycin (Sm)/novobiocin (Nb) was used to select against donors. Exconjugants carrying pWBG749e and pLI50 were selected for using Em and Cm, respectively.

For analysis of staphylococcal plasmid sequences, the NCBI RefSeq database was queried with the term ‘Staphylococcus[Organism] AND plasmid[Title]’. Retrieved sequences were added to an existing collection of *S. aureus* plasmid sequences (11). Duplicates were identified by sorting sequences by name and accession number and by using Elimdupes (http://hcv.lanl.gov/content/sequence/ELIMDUPES/elimdupes.html). Duplicate sequences and sequences >60 kb were discarded. This produced a collection of 360 unique plasmid sequences as of January 10th 2013 (Supporting Dataset S1). The sequences were used to create custom BLASTN databases using standalone BLAST version 2.2.20 (36). To identify *oriT* sequences, the region from DNA motif AR3 to the core sequence was used as a query and each sequence was manually inspected before inclusion. Identified sequences with novel IR2 repeats were used in subsequent searches and this process was iterated until no new IR2 repeat variants were identified. Once *oriT* sequences were categorised, additional BLASTN databases were created of plasmid sets with and without *oriT* sequences. TBLASTN searches for cadmium resistance and mobilisation loci on *S. aureus* plasmids were carried out using CadA (GI:335749909), CadD (GI: 695199049), MobA (GI:212383462) and Pre (GI:60392927) as query sequences. TBLASTN searches for antimicrobial-resistance loci on pWBG749-like contigs in the Orange County MRSA dataset were carried out using BlaZ (SAP030A_004), AacA-AphD (GI:618846397), VanA (YP_003864126), VanB (GI:618846277), MphBM (GI:3892644), CAT (GI:1015408) and TetM (GI:302750286) as queries. All BLAST hits were manually inspected for false positives.

Single-stranded DNA (ssDNA) structure predictions were carried out using Mfold (37) and predicted structures were redrawn using VARNA (38). DNA alignments of the *oriT* sequences were carried out using T-Coffee in rcoffee mode with default settings (39,40) or Clustal W (41) and redrawn using Jalview (42). Circular BLASTN alignments were carried out using BLASTN (36) in ‘ungapped’ mode and displayed using BRIG (43). Tree construction was carried out using PhyML software using a neighbour-joining starting tree with otherwise default settings. Construction of a maximum credibility tree using BEAST (using a random local-clock model and otherwise default parameters) and TreeAnnotator (44) produced a tree that supported all major nodes of the PhyML-produced tree (not shown).

## RESULTS

### Identification of the pWBG749 *oriT* sequence

Sequence comparisons of pWBG745, pWBG748, pWBG749 and pBRZ01 revealed that all these plasmids carried a conserved ∼24 kb cluster (Supplementary Figure S1) carrying 24 open-reading-frames (ORFs), encoding proteins with likely roles in conjugation (Supplementary Table S3) (25). Proteins encoded by genes in this cluster were renamed staphylococcal mobilisation plasmid proteins SmpA-SmpX. PSI-BLASTP queries using SmpP (SAP031A_037) revealed it showed similarity to relaxases on conjugative plasmids of *Bacillus thuringiensis* MC28 (pMC189) and *Lactococcus gavieae* (pGL5) and the vancomycin-resistance plasmids pMG2200 of *Enterococcus faecalis* and pHTβ of *E. faecium* (45–48) (Supplementary Figure S2). Homologues of 17/24 of the Smp proteins were identified amongst these plasmids (Supplementary Table S3). SmpP carried N-terminal sequence motifs common to the MOB_MG_ family of relaxases (46,48), including two conserved tyrosine residues and a histidine-triad motif likely required for divalent cation binding (Supplementary Figure S2). Analysis of SmpP using CONJscan (49), which scans a database of hidden-markov-model profiles created from a curated database of conjugation proteins, placed SmpP in the large MOB_P_ family of relaxases (E = 4 × 10^−47^).

**Figure 1.**
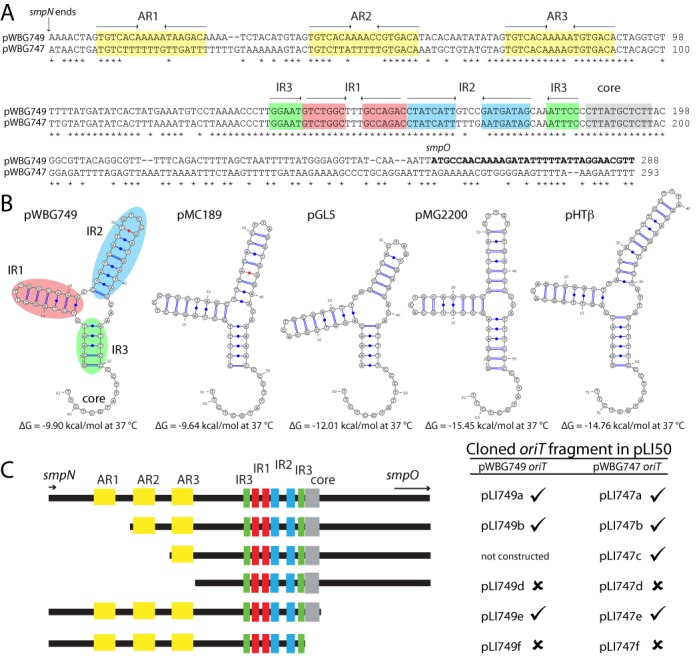
Conserved repeat structure of the pWBG749 *oriT*. (**A**) Sequence alignment of DNA between *smpN* and *smpO* on pWBG749 and the *oriT* sequence identified on mobilisable plasmid pWBG747 (25). Both regions were cloned into pLI50 and used in conjugative mobilisation experiments in panel (C). Repeat structures are highlighted with colours that correspond to the structure predictions in *B* and constructed clones in panel (C). (**B**) Mfold (37) predictions of the ssDNA structure formed adjacent to the core sites found on pWBG749, pMC189, pGL5, pMG2200 and pHTβ. Estimated Gibbs free-energy values for each structure are shown below. Mfold-predicted non-classical base-pairing is shown in red. (**C**) Conjugative mobilisation of the pWBG749 and pWBG747 *oriT* sequence-carrying constructs and delineation of the minimal *oriT* sequence. Cloned *oriT* fragments (a-f) are depicted on the left and the outcome of mobilisation experiments (Table 1) is indicated on the right (as ticks or crosses) for clones carrying pWBG749 or pWBG747-derived *oriT* sequences.

*oriT* DNA sequences are commonly located upstream of their cognate Mob gene and usually comprise at least one hairpin-forming inverted-repeat (IR) sequence located 5′ to a ‘core’ region containing the DNA cleavage site *nic*. Core sequences are often conserved between divergent *oriT* sequences (50), presumably due to evolutionary constraints on the catalytic site of the Mob protein. Comparison of the nearest non-coding DNA located upstream of the Mob genes on pWBG749, pMG2200, pMC189, pHTβ and pGL5 revealed that each region carried the sequence 5′-CTTATGCTCTT-3′ (hereafter referred to as the *oriT* core). A structurally comparable configuration of IR sequences was identified adjacent to the core site on each plasmid (IR1, IR2 and IR3). Mfold secondary-structure prediction (37) indicated each region could adopt a similar ssDNA branched-hairpin structure adjacent to the core sequence, despite a lack of repeat-sequence similarity (Figure 1B). Three additional IR sequences (accessory repeats AR1-AR3) were located upstream of IR1-IR3 on pWBG749 (Figure 1A). pMG2200 and pHTβ carry distinct repetitive sequences in the same region (46,48).

The putative *oriT* region identified between the *smpN* and *smpO* genes (Figure 1A) was amplified by polymerase-chain-reaction (PCR) and cloned into the non-mobilisable shuttle vector pLI50 (carrying Cm resistance) (51), producing pLI749a. pLI50 and pLI749a were introduced into *S. aureus* strain RN4220 by electroporation. Plasmid pWBG749e (a pWBG749 derivative carrying Em resistance (25)) was introduced into each strain by conjugation and the resulting exconjugants were used as donors in mobilisation experiments. pLI749a, but not pLI50, was able to be transferred by conjugation to WBG4515 (Sm/Nb-resistant derivative of RN450) at an average frequency of 6.9 × 10^−7^ recipients per donor, roughly 40-fold lower than the average conjugation frequency of pWBG749e (2.8 × 10^−5^) from the same donor (Table 1). No pLI50/pLI749a transfer was observed from donors lacking pWBG749e. To estimate the frequency of plasmid cotransfer, Cm/Sm-resistant WBG4515 exconjugants carrying pLI749a were subcultured on media containing Em/Cm/Sm. This revealed 43–75% (85/126 colonies from 2 experiments) of exconjugants carrying pLI749a also carried pWBG749e. Since pLI49a was able to transfer alone to WBG4515, this suggested that the observed mobilisation of pLI749a was not due to *oriT-*mediated recombination/cointegration with pWBG749e. To discount the possibility that recombination between the *oriT* sites on pWBG749e and pLI749a was responsible for the plasmid cotransfer, DNA was extracted from an Em/Cm/Sm/Nb-resistant exconjugant and the pLI749a *oriT* was PCR-amplified and sequenced using pLI50-specific primers, which would not produce a PCR product if recombination had occurred between the *oriT* sites on each plasmid. In summary, these experiments confirmed that the region between *smpN* and *smpO* encompassed the pWBG749 *oriT* and that carriage of this *oriT* conferred the ability to be mobilised by pWBG749.

**Table 1.** Mobilisation of plasmids carrying cloned *oriT* fragments from pWBG749 and pWBG747

		Transfer frequency^a^	
Plasmid name	*oriT* source	pWBG749e	pLI50 clone
pLI50	Vector-only control	5.0 × 10^−6^	Not detected
		1.3 × 10^−5^	Not detected
		2.6 × 10^−5^	Not detected
pLI749a	pWBG749	1.1 × 10^−5^	1.4 × 10^−6^
		1.0 × 10^−5^	4.9 × 10^−7^
		6.3 × 10^−5^	1.7 × 10^−7^
pLI749b	pWBG749	1.3 × 10^−5^	3.1 × 10^−7^
		3.3 × 10^−5^	2.1 × 10^−7^
		8.7 × 10^−5^	1.0 × 10^−7^
pLI749d	pWBG749	3.7 × 10^−6^	Not detected
		4.9 × 10^−5^	Not detected
		1.0 × 10^−4^	Not detected
pLI749e	pWBG749	9.7 × 10^−6^	1.1 × 10^−6^
		1.7 × 10^−5^	6.2 × 10^−7^
		5.8 × 10^−5^	2.6 × 10^−7^
pLI749f	pWBG749	2.5 × 10^−5^	Not detected
		2.1 × 10^−5^	Not detected
		1.4 × 10^−4^	Not detected
pLI747a	pWBG747	1.1 × 10^−5^	1.4 × 10^−7^
		1.0 × 10^−5^	6.0 × 10^−7^
		8.9 × 10^−6^	8.0 × 10^−8^
pLI747b	pWBG747	2.4 × 10^−5^	4.7 × 10^−7^
		2.2 × 10^−5^	5.4 × 10^−7^
		2.0 × 10^−5^	3.9 × 10^−7^
pLI747c	pWBG747	8.4 × 10^−6^	6.0 × 10^−7^
		2.3 × 10^−5^	3.7 × 10^−7^
		2.0 × 10^−5^	1.9 × 10^−7^
pLI747d	pWBG747	1.7 × 10^−5^	Not detected
		1.8 × 10^−5^	Not detected
		8.4 × 10^−5^	Not detected
pLI747e	pWBG747	1.5 × 10^−5^	2.4 × 10^−7^
		3.1 × 10^−5^	8.8 × 10^−7^
		4.5 × 10^−5^	1.0 × 10^−6^
pLI747f	pWBG747	2.1 × 10^−5^	Not detected
		3.7 × 10^−5^	Not detected
		1.9 × 10^−5^	Not detected

^a^Per-donor frequencies of individual mating experiments.

### The minimal pWBG749 *oriT* region is conserved on diverse mobilisable plasmids and confers the ability to be mobilised

pWBG749 mobilises the transfer of several genetically distinct plasmids that lack identifiable Mob sequences (25). BLASTN searches of plasmid sequences of pWBG744, pWBG747, pWBG756, pWBG761 and pWBG762, revealed that they each carried a sequence sharing 85–86% nucleotide identity with pWBG749 *oriT* (Supplementary Figure S3). To investigate if the identified putative *oriT* sequences (designated ‘OT49’ subfamily *oriT* sequences; see explanation in following paragraphs) enabled mobilisation by pWBG749, the *oriT* sequence from each plasmid was cloned into pLI50 and the resulting constructs pLI744, pLI747, pLI762–49, pLI761a and pLI756b were used in mobilisation experiments. Each construct was capable of being mobilised by pWBG749e at a rate similar to that of pLI749a (Table 2). Next the minimal *oriT* regions of pWBG749 and pWBG747 were defined by cloning products of the *oriT* that each lacked one or more sequence motifs highlighted in Figure 1A. Clones lacking AR1 or AR2 were mobilised at rates similar to pLI749a, but transfer was undetectable from clones lacking all three AR repeats (Table 1). Comparison of the AR sequences (Supplementary Figure S3) carried by mobilisable plasmids revealed that only AR3 was strongly conserved, consistent with the non-essentiality of AR1 & AR2 for mobilisation. Clones lacking DNA downstream of the *oriT* core exhibited similar transfer rates to that of pLI749a, while clones lacking the *oriT* core were unable to be mobilised (Figure 1C and Table 1). In summary, these experiments confirmed that carriage of the ∼126-bp minimal *oriT* by diverse antimicrobial-resistance plasmids conferred their ability to be mobilised by pWBG749.

**Table 2.** pWBG749e-dependent mobilisation of *oriT* sequences cloned from diverse non-conjugative antimicrobial-resistance plasmids

		Transfer frequency^a^	
Plasmid name	Cloned *oriT* (group designation)^b^	pWBG749e	pLI50 clone
pLI744	pWBG744 (OT49_F)	6.9 × 10^−5^	6.4 × 10^−7^
pLI747	pWBG747 (OT49_M)	7.3 × 10^−5^	4.7 × 10^−7^
pLI762–49	pWBG762 (OT49_E)	7.3 × 10^−5^	1.5 × 10^−6^
pLI761a	pWBG761 (OT49_G)	1.2 × 10^−4^	8.1 × 10^−7^
pLI756b	pWBG756 (OT49_N)	6.8 × 10^−5^	2.8 × 10^−7^
pLI762–45	pWBG762 (OT45)	3.5 × 10^−5^	Not detected
pLI762-UNa	pWBG762 (OTUNa)	6.4 × 10^−5^	Not detected
pLI50		7.9 × 10^−5^	Not detected

^a^Transfer frequencies are presented as per-donor frequencies and are the average of three experiments.

^b^See text and Figure 3 and Supplementary Figure S4 for explanation *oriT* group designations.

### pWBG749-like *oriT* sequences are found on 53% of *Staphylococcus aureus* plasmid sequences

**Figure 2.**
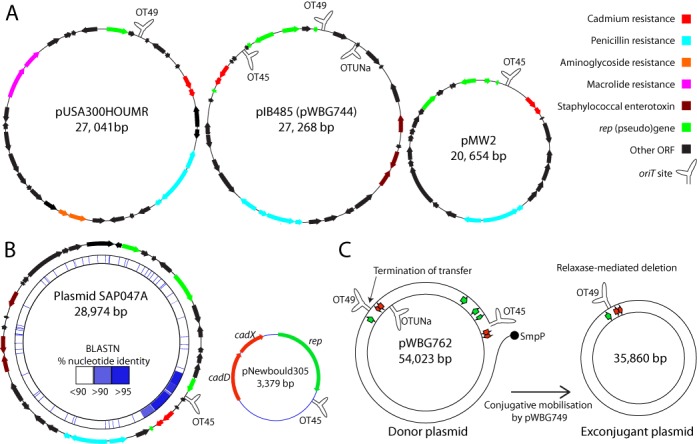
Plasmids carrying pWBG749-family *oriT* sequences. (**A**) Diagrams of representatives of the three most prevalent families of large staphylococcal plasmids, pMW2, pUSA300HOUMR and pIB485 (5), showing the relative positions of resistance genes, replication genes and *rep* gene fragments, staphylococcal-enterotoxin genes and *oriT* sites (see key on left, colour coding is also applicable to part B). Please see text, Figure 3 and Supplementary Figure S4 for explanation of *oriT* subfamily ‘OT’ designations: OT49, OT45 and OTUNa. (**B**) Diagrams of plasmids SAP047A and pNewbould305 (not to scale). pNewbould305 carries cadmium-resistance genes *cadDX*, a *rep* gene and an OT45-type *oriT*. The pNewbould305 sequence is >90% identical to a region on plasmid SAP047A, suggesting this region represents an integration of a pNewbould305-like plasmid (BLASTN comparison was made using BRIG (43)). (**C**) pWBG762 carries three *oriT* sequences and two *cadDX* loci as well as several *rep* gene fragments, suggesting multiple acquisitions of *oriT-*carrying cadmium-resistance RCR plasmids. The schematic illustrates the possible mechanism of the pWBG749-mediated deletion that occurred following conjugative mobilisation of pWBG762. Transfer presumably initiated at the OT45 site and terminated at the OT49 site.

Identified *oriT* sequences (spanning from AR3 to the core) were used as queries in BLASTN searches against a database of 360 non-identical staphylococcal-plasmid sequences collected from GenBank (Supporting dataset S1). Of 280 *S. aureus* plasmids, 147 (53%) carried sequences resembling the pWBG749 *oriT*. These included the three most prevalent large (>20 kb) plasmid families: pMW2, pUSA300HOUMR and pIB485 (pWBG744) (Figure 2A). In a large-scale analysis of *S. aureus* plasmids isolated between 1940 and 2011, these three plasmid families represented 49% of all typed plasmids greater than 20 kb (5). In contrast only 9% of plasmids (7/80) isolated from other staphylococcal species carried the *oriT* sequence (Supporting dataset S1). *oriT* sequences were found on RCR plasmids (22 plasmids ranging from 2.8 to 4.4 kb) and larger theta-replicating plasmids (132 plasmids ranging from 14.6 to 57.9 kb). TBLASTN searches failed to identify ORFs with similarity to Mob (52) or Pre (53) relaxase genes on the *oriT*-carrying RCR plasmids. Intact Mob or Pre ORFs were identified on only four theta-replicating plasmids (pGO1, SAP016A, SAP106A and pPM1). This general absence of Mob/Pre genes on *oriT*-carrying plasmids is consistent with the proposed relaxase-*in trans* mechanism of pWBG749-mediated mobilisation. Of the larger *oriT*-carrying plasmids, 42 plasmids harboured two copies of the *oriT* sequence and 15 harboured three copies. Inspection of the BLASTN output of *oriT-*sequence queries revealed that plasmids encoding two *oriT* sequences invariably carried them on opposite DNA strands, while all plasmids encoding three *oriT* never carried all three on the same strand. In total, 229 sequences closely resembling the pWBG749 *oriT* were identified on 154 staphylococcal-plasmid sequences in the dataset.

*oriT* sequences identified on RCR plasmids were consistently located downstream of the replication genes (see Figure 2B for example), but the association was not specific to a single *rep* -gene family (pWBG1773, pACK5 and pPI-2 carry *rep*_13_-family, the remainder carry *rep*_25_ (11)). *rep* pseudogenes and gene fragments were also identified adjacent to *oriT* sequences on most theta-replicating plasmids (see Figure 2A for example), suggesting that acquisition of *oriT* sequences by larger plasmids had occurred through cointegration with RCR plasmids. A clear example of a cointegration event involved the 29-kb penicillinase and enterotoxin-encoding plasmid SAP047A, isolated from human synovial fluid isolate *S. aureus* SAP047 (5). SAP047A contained a region sharing >90% nucleotide identity with the cadmium-resistance and *oriT-*carrying RCR plasmid pNewbould305, from bovine mastitis strain *S. aureus* Newbould 305 (54) (Figure 2B). Cadmium-resistance loci *cadDX* and *cadAC* were also frequently encoded adjacent to *oriT* sequences on large plasmids (Figure 2). TBLASTN searches revealed that 79% (121/154) of *oriT*-encoding plasmids carried copies of either CadA (41/154) or CadD (100/154), compared to only 9% for plasmids not encoding an *oriT* (18/206). These observations strongly suggest that acquisition of *oriT* sequences by large *S. aureus* plasmids has occurred through integration of RCR plasmids carrying cadmium-resistance loci (similar to pNewbould305 (Figure 2B)) into larger antimicrobial-resistance and virulence-gene plasmids.

Whole-genome sequencing data for 397 clinical *S. aureus* isolates was recently released by the MRSA Orange County initiative of the Broad Institute (broadinstitute.org) (not included in plasmid database in Supporting dataset S1). BLASTN of this dataset using the pWBG749 sequence as a query identified 24 contigs with contiguous nucleotide similarity across the entire pWBG749 conjugation cluster (Supplementary Figure S1 and Supporting dataset S3). These putative pWBG749-family plasmids were carried by strains isolated from blood, wounds and sputum. Astonishingly, 736 copies of the *oriT* sequence were identified across all contigs for the 397 genomes. Thus pWBG749-like plasmids are present in a minority of clinical *S. aureus* isolates, but pWBG749-like *oriT* sequences appear to be abundant.

### *oriT* sequences carry different IR2 sequences that correspond to distinct *oriT* sequences carried by five pWBG749-like conjugation-gene clusters

**Figure 3.**
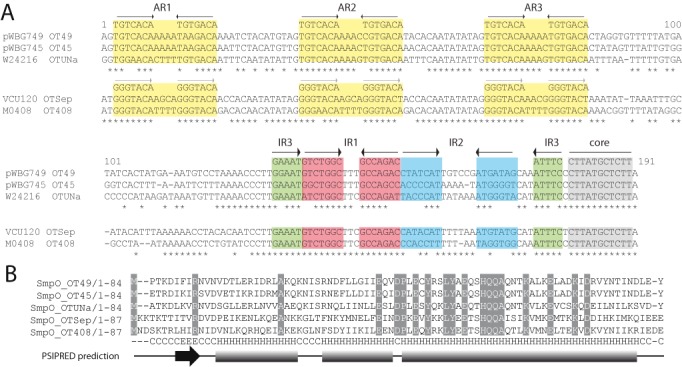
Comparison of OT49, OT45, OTUNa, OTSep and OT408 pWBG749-subfamily *oriT* and SmpO sequences. (**A**) The intergenic regions upstream of *smpO* on pWBG749 and pWBG745 were aligned (using Clustal W (41)) with the corresponding *oriT* region identified in the shotgun genome sequence of *S. aureus* W24216 (GI:582299942). Corresponding regions on putative conjugation clusters identified in *S. epidermidis* VCU120 (GI:418616860) and *S. aureus* M0408 (GI:477787193) were aligned separately. AR and IR repeats are highlighted as in Figure 1. The consensus for AR, IR and core sequences are shown above each alignment. OTSep and OT408 AR sequences are distinct from those located on OT49, OT45 and OTUNa. Each *oriT* family has a distinct IR2 sequence highlighted in blue. (**B**) A T-coffee alignment (mcoffee mode) of the SmpO sequences identified on pWBG749-family conjugation clusters. Each SmpO gene is labelled by its corresponding ‘OT’ grouping. Completely conserved residues are shaded. The output of a PSIPRED secondary-structure prediction from the alignment of sequences is shown below (H = helix, C = coil, E = strand). The first half of each protein resembles the classical β-sheet-helix-helix structure of ribbon-helix-helix proteins.

The observation that several plasmids carried multiple *oriT* sequences suggested that each *oriT* might be functionally distinct. Closer inspection revealed that when multiple *oriT* sequences were carried on a single plasmid they often contained different IR2 sequences. Sequence alignment of the region encompassing AR3, IR1, IR2, IR3 and the core was carried out for all *oriT* sequences identified in our plasmid database using T-coffee in rcoffee (RNA) mode, to account for ssDNA structural motifs (Supplementary Figure S4). Tree construction using PhyML (55) clustered the 53 unique *oriT* sequences into three major clades with >91% bootstrap support (Supplementary Figure S5). Subclades with lower bootstrap support were supported by a maximum credibility tree constructed using Markov Chain Monte Carlo software BEAST and TreeAnnotator using the same DNA alignment (not shown). Further inspection of the DNA sequence alignments within each clade revealed that there were at least five distinct IR2 sequence variants, while the repeat sequences of IR1, IR3 and the core were near-identical between all the sequences. Two of the distinct *oriT* IR2 variants (named OT49 and OT45) corresponded with IR2 motifs within *oriT* sequences on conjugative plasmids pWBG748/pWBG749 and pWBG745/pBRZ01, respectively, while a further three IR2 motifs matched *oriT* sequences located upstream of SmpO-gene homologues on pWBG749-like conjugation clusters found in the genome sequences of *S. aureus* M0408 (named OT408), *S. epidermidis* (named OTSep) and *S. aureus* W24216 (named OTUNa) (Figure 3A). Of the pWBG749-like contigs present in the Orange County MRSA dataset, 3 were OT49 and 21 were OT45 type, but OT49 *oriT* sites were five times more abundant than OT45 sites across the dataset (502 versus 100, (Supporting dataset S3)). OT45, OT49 and OTUNa all carried AR sites resembling those of pWBG749, while OTSep and OT408 *oriT* sites contained a different set of AR repeats in the same positions (Figure 3A). Interestingly, OTUNa sites were only identified on larger plasmids and in each case they were located directly upstream of a truncated copy of *smpO*, suggesting that acquisition of the OTUNa sites had occurred through direct capture of the *oriT*-*smpO* region from a conjugative plasmid.

Inspection of plasmids carrying multiple *oriT* sequences revealed that most did not carry copies from the same *oriT* subfamilies [58/67]. The most common configuration for plasmids carrying three *oriT* sequences was OT49/OT45/OTUNa [13/15], while for those carrying two *oriT* sequences it was OT49/OT45 [27/42]. Plasmids carrying the combinations OT45/OTUNa [7], OT49/OT49 [7] OTSep/OTUNa/OTUNa [1], OT45/OT49/OT49 [1], OTSep/OTUNa [1] and OTSep/OT408 [1] were also observed. Therefore, assuming *oriT* acquisition is a random process, most plasmids appear to have preferentially maintained or acquired *oriT* sequences of different subfamilies, further indicating that the *oriT* sites are not functionally equivalent.

### *smpO* acts as an *oriT* subfamily specificity factor

Plasmids pWBG749 and pWBG745 are 99% identical over 93% of their nucleotide sequences (25), but carry *oriT* sequences from different subfamilies (OT49 and OT45, respectively) (Supplementary Figure S1 and Figure 3A). pMW2 and pWBG757 each carry a single OT45 *oriT* and are not mobilised by pWBG749 (25). pWBG762 carries three *oriT* sequences (OT49, OT45 and OTUNa) and a plasmid carrying the pWBG762 OT49 *oriT* (pLI762–49) was mobilised by pWBG749. The OTUNa and OT45 sites from pWBG762 and the *oriT* of pWBG745 were cloned into pLI50 and used in mobilisation experiments. Neither of the resultant clones were able to be mobilised by pWBG749e (Table 1) indicating that pWBG749 was not able to efficiently mobilise plasmids carrying OT45 or OTUNa *oriT* sites. This suggested that conjugative plasmids with different IR2 sequences, like pWBG749 and pWBG745, may only be able to mobilise plasmids carrying *oriT* sequences of the same subfamily, i.e. with matching IR2 sequences.

A comparison of the SmpP sequences of pWBG749 and pWBG745 revealed they differed by only 3 amino acids (out of 382). To investigate if the SmpP genes of pWBG749 and pWBG745 (*smpP*_49_ *and smpP*_45_, respectively) were responsible for the discrimination of OT49 and OT45 *oriT* sites by pWBG749, the *oriT-smpO-smpP* regions of both plasmids were cloned into pLI50, producing pKY9TOP and pKY5TOP. pWBG749 mobilised both these clones at rates similar to those of pLI749a, indicating that the presence of pWBG745-encoded *smpO* or *smpP* enabled pWBG749-mediated mobilisation of OT45 sites (Table 3). To test if the *smpP* genes were responsible for *oriT* specificity, the *smpP* gene of each clone was swapped, producing pKY9TO5P and pKY5TO9P. Both clones were mobilised at similar rates, clearly indicating SmpP was not the specificity factor required for discrimination between OT49 and OT45 *oriT* sites. Moreover, this result implied that it was the presence of pWBG745 *smpO* (*smpO*_45_) on pKY5TO9P that enabled mobilisation of the OT45 site by pWBG749. To test this hypothesis, a clone carrying only the *oriT-smpO* region of pWBG745 was constructed, producing pKY5TO. This clone was mobilised by pWBG749 at levels similar to pLI749a, confirming that the presence of pWBG745-encoded *smpO* enabled the mobilisation of the OT45 *oriT* sequence by pWBG749. Thus the SmpO proteins of pWBG749-family plasmids likely act as specificity factors for the *oriT*, possibly through direct recognition of the IR2 sequence. A comparison of the SmpO sequences encoded on pWBG749 and pWBG745 revealed that they shared 86% amino-acid identity and have diverged mostly in their N-terminal regions (predicted β-sheet region) (Figure 3B).

**Table 3.** Mobilisation of an OT45 *oriT* by pWBG749e

			Transfer frequency^a^	
Plasmid name	Cloned *oriT* (OT group designation)^b^	Additional genes cloned^c^	pWBG749e	pLI50 clone
pLI749a	pWBG749 (OT49)		4.0 × 10^−5^	3.9 × 10^−6^
pKY9TOP	pWBG749 (OT49)	*smpO*_49_*smpP*_49_	1.7 × 10^−5^	2.1 × 10^−6^
pKY9TO5P	pWBG749 (OT49)	*smpO*_49_*smpP*_45_	2.3 × 10^−5^	2.8 × 10^−6^
pKY5T	pWBG745 (OT45)		2.4 × 10^−5^	Not detected
pKY5TOP	pWBG745 (OT45)	*smpO*_45_*smpP*_45_	1.5 × 10^−5^	8.5 × 10^−6^
pKY5TO9P	pWBG745 (OT45)	*smpO*_45_*smpP*_49_	2.4 × 10^−5^	9.0 × 10^−6^
pKY5TO	pWBG745 (OT45)	*smpO*_45_	1.3 × 10^−5^	6.6 × 10^−6^
pLI50	empty vector		2.3 × 10^−5^	Not detected

^a^All transfer frequencies are presented as per-donor frequencies and are the average of three experiments.

^b^See text, Figure 3 and Supplementary Figure S4 for explanation *oriT* group designations.

^c^Subscript ‘49’ refers to genes cloned from pWBG749, subscript ‘45’ refers to genes cloned from pWBG745.

### pWBG749 stimulates recombination between OT49 and OT45 core sites

Relaxase-mediated recombination between same-strand *oriT* sequences has been documented for plasmids and integrative and conjugative elements (ICEs) (53,56–61). Relaxase cleavage at the first *oriT* and ligation with the second *oriT*, produces a deletion of DNA outside the transferred region, delineated by the *oriT* core site. In our analysis of plasmids carrying multiple *oriT* sequences, we observed a complete absence of same-strand *oriT* sequences on plasmids carrying two *oriT* sequences. Because of this bias we suspected that relaxase-mediated deletion of DNA between same-strand encoded *oriT* pairs might have removed many same-strand *oriT* pairs from the plasmid population. Analysis of the mobilisable plasmid pWBG762 revealed it carried 3 *oriT* sites and that the OT49 and OT45 sites were located on the same strand. pWBG762 DNA was isolated from 11 exconjugants following mobilisation by pWBG749e (25). Restriction analysis revealed that one of the eleven exconjugants carried a pWBG762 plasmid with a unique EcoRI restriction pattern and an apparent size of 35.9 kb, rather than the expected 54.0 kb (Supplementary Figure S6). The size reduction in pWBG762 was consistent with a deletion between the OT45 and OT49 *oriT* sites. Primers flanking the OT45 and OT49 sites successfully amplified the predicted deletion site. Sequencing confirmed recombination had occurred between the *oriT* core sites of the OT49 and OT45 *oriT* sequences, leading to a deletion of the intervening DNA (including the OTUNa site) (Supplementary Figure S6). The nucleotide polymorphisms between the OT45 and OT49 *oriT* sequences (Supplementary Figure S6) and mobilisation experiments with truncated *oriT* sequence clones (Figure 1), together delineated the relaxase *nic* site to be within a 16-bp sequence (5′-CCTTATGCTCTTACGG-3′) containing the 11-bp sequence conserved on divergent plasmids carrying MOB_MG_-family Mob proteins (Figure 1B and Supplementary Figure S6).

## DISCUSSION

It is well established that conjugative plasmids can mobilise plasmids that are not self-transmissible, but the vast majority of documented mobilisable plasmids encode distinct Mob protein(s) and carry distinct *oriT* (17). The Mob proteins of mobilisable plasmids are able to exploit the T4SS encoded by conjugative plasmids through interaction with the VirD4/TraG coupling-protein component of the mating pore (62,63). In this study we confirmed that pWBG749 is capable of mobilising plasmids that lack Mob genes and that this ability is due to these mobilisable plasmids carrying *oriT* sites that resemble that of pWBG749. This indicates that the pWBG749 relaxasome acts *in trans* on antimicrobial-resistance plasmids and mobilises their recruitment to the mating pore. Most importantly, *oriT* sequences resembling that of pWBG749 were found on most *S. aureus* plasmids including all three of the most prevalent large *S. aureus* antimicrobial-resistance plasmids, pIB485, pMW2 and pUSA300HOUMR (5). This supports the hypothesis that this relaxase-*in trans* mechanism is a dominant mechanism of plasmid mobilisation in *S. aureus*. It is notable that the recently emerged vancomycin-resistant strain BR-VRSA (9,64), as well as carrying the pWBG749-family conjugative vancomycin-resistance plasmid pBRZ01, also carries a plasmid almost identical to pMW2 (pHMPREF1625_1, GI:618846309). Since both plasmids carry an OT45 subfamily *oriT*, we would predict that pBRZ01 mobilised the transfer of the pMW2 plasmid into this strain background during evolution of BR-VRSA and that BR-VRSA is capable as acting as a conjugative donor of pMW2.

The pWBG749 *oriT* sequence was initially identified through the comparison of MOB_MG_ conjugative plasmids pMC189, pGL5, pMG2200 and pHTβ (45–48). The *oriT* sequences, despite having little sequence similarity, are all predicted to form a branched hairpin structure, suggesting this is a conserved structural feature of the cognate *oriT* sequences of MOB_MG_ Mob proteins. Mob relaxases bind DNA hairpin structures adjacent to the *oriT* core and these hairpins have roles in recognition, replication and plasmid recircularisation (65–69). pWBG749-family plasmids have diverged into five distinct subfamilies, each carrying a different IR2 variant of the *oriT*. pWBG749 was unable to mobilise plasmids carrying OT45 or OTUNa *oriT* sequences, but pWBG749 mobilisation of an OT45 *oriT-*carrying construct was enabled in the presence of the pWBG745-encoded *smpO*. This suggested that SmpO acts as a specificity factor for the *oriT*, possibly through binding the IR2 sequence. Comparison of the SmpO proteins of pWBG749 and pWBG745 revealed that unlike their near-identical SmpP proteins, their SmpO proteins have diverged more significantly in their N-terminal sequence (Figure 3B). Secondary-structure prediction for the SmpO proteins indicates their N-terminal half contains a sheet-helix-helix structure and database PSI-BLAST searches retrieve weak hits to the ribbon-helix-helix (RHH) family of DNA-binding proteins. RHH proteins have been previously associated with relaxasomes and regulation of conjugative transfer (70–72). The N-terminal regions of RHH protein dimers form an antiparallel β-sheet that fits into the major groove of the DNA and the N-terminal sequence contributes almost exclusively to the decoding of the DNA-binding site (73). Therefore the sequence divergence within this region for the SmpO proteins of pWBG749 and pWBG745 fits with their differential *oriT* specificity. Interestingly, apart from the region encompassing *oriT–smpO*, pWBG745 and pWBG749 are almost identical at the nucleotide level within the predicted conjugation cluster (Supplementary Figure S1). This might suggest that the *oriT*-*smpO* region of pWBG745 (or conversely pWBG749) may have been recently acquired, enabling the evolved plasmid to recognise OT45 sites or avoid exploitation by non-conjugative plasmids carrying OT49 sites. In contrast, the *S. aureus* W24216 SmpO sequence shows only 49% and 46% amino-acid identity to SmpO genes of pWBG749 and pWBG745 (respectively), despite being encoded downstream of an OTUNa *oriT* sequence that closely resembles OT45 (Figure 3 and Supplementary Figure S4). Regardless of the evolutionary reasons for this divergence in *oriT* specificity, the observation that several plasmids have captured multiple *oriT* sequences from these different subfamilies implies that they have benefited from mobilisation by pWBG749-family plasmids capable of mobilising each *oriT* subfamily during their evolutionary history.

pWBG749 was unable to mobilise OT45 sites in the absence of pWBG745 *smpO* (Table 3), but it was able to stimulate recombination between OT45 and OT49 sites on pWBG762 during mobilisation (Figure 2C and Supplementary Figure S6). During conjugation, Mob relaxases are covalently attached to the 5′ phosphate on the nicked DNA strand such that ssDNA downstream of the *oriT* core is piloted through the T4SS by the relaxase. RCR replaces the displaced ssDNA, after which a Mob molecule mediates a second cleavage at the reconstituted *oriT* site (16). The relative orientation of the OT45 and OT49 sites on pWBG762 and the subsequent deletion that occurred following transfer, indicates transfer initiated at the OT45 site and terminated at the OT49 site. Initially this appears to conflict with our previous observation that pWBG749 could not mobilise OT45 sites in the absence of pWBG745 *smpO*. This indicates that recognition of IR2 is not strictly essential for recognition and cleavage by the SmpP relaxase. For the plasmid R388 relaxase TrwC, only the *oriT* core sequence is strictly required for recognition and cleavage, but the hairpin sequence 5′ adjacent to the core is required for TrwC binding on a ssDNA target and is essential for efficient transfer (74). It is possible that recognition of IR2 by SmpO/SmpP is only strictly required for termination of rolling-circle replication or plasmid recircularisation. If this is the case then pWBG749 family plasmids may have an ability to initiate transfer or facilitate recombination of any plasmid that carries the conserved MOB_MG_ *oriT* core site identified in this study.

A curious observation in our analyses of the *oriT* sequences was their genetic linkage to cadmium-resistance loci and RCR-Rep genes. Comparisons between plasmids like pNewbould305 and larger *oriT*-carrying plasmids such as SAP047A reveal a clear cointegration event between the ancestors of these plasmids, tentatively explaining the association of *oriT* sequences with RCR Rep genes (Figure 2). From inspection of plasmids pIB485 and pWBG762, it seems that the process of plasmid cointegration has occurred repeatedly during evolution (Figure 2). For these examples it is unclear if this process reflects selection for heavy-metal resistance or an ability to be mobilised by multiple pWBG749-like plasmids, or perhaps both. There was a clear exception to this genetic linkage between cadmium-resistance loci and *oriT* sequences: The OTUNa *oriT* sequences were not frequently located adjacent to cadmium-resistance loci, but were all located adjacent to an *smpO* pseudogene. This suggests that this sequence was obtained directly through recombination with a conjugative plasmid and supports the hypothesis that *oriT* sequences have been the focus of selection, at least in this case.

Despite the resounding genetic footprints left by plasmids of the pWBG749 family in the form of *oriT* sequences, there is a paucity of pWBG749-like plasmids and pGO1/pSK41 plasmids in sequence databases (5). The lack of conjugation genes in extant *S. aureus* genomes seems to imply that conjugation events are rare or that most apparent horizontal gene transfer events may have occurred ancestrally. Indeed, the prevalent pIB485 family of plasmids, which carries OT49, OT45 and OTUNa sites, existed in its present form prior to 1949 (5), indicating selection for capture of *oriT* sequences and mobilisation by pWBG749-like plasmids occurred prior to the widespread of use of antimicrobials. Nevertheless, pWBG749-family plasmids are extant in *S. aureus* populations and *oriT* sequences are abundant, as revealed by our analysis of the Orange County dataset. We speculate that conjugative plasmids may be more prevalent in *S. aureus* populations than is reflected by current sequence data, possibly due to biases inherent in the sequencing of disease-causing isolates. Clinical isolates have likely experienced population bottlenecks, periods of diversification and rapid clonal expansion during infection and antimicrobial treatment (75,76). The fitness cost of conjugative-plasmid carriage (22), coupled with a lack of direct selection for their maintenance may have led to loss of pWBG749 plasmids in the most prevalent pathogenic isolates. While antimicrobial-resistance carrying derivatives of pWBG749 and pWBG745 have been identified (pWBG748 (25) and pBRZ01 (9), respectively), these are an exception. Of the 24 pWBG749-family contigs detected in the MRSA Orange County sample, genetic determinants for aminoglycoside, macrolide and vancomycin resistance were not detected and only one contig (LAMC0050) carried genes for β-lactamase production (Supporting dataset S3). It is of note, that unlike pBRZ01, plasmids pWBG745, pWBG748 and pWBG749 were found in CA-MRSA strains isolated from asymptomatic carriers, demonstrating that healthy individuals can be reservoirs of pWBG749-family plasmids.

It is clear from the abundance of pWBG749-like *oriT* sequences that the *in trans* mechanism of mobilisation is a dominant mechanism of mobilisation in *S. aureus*. So why is there such a paucity of evidence for this mechanism in other species? One explanation may be that the relaxase-*in trans* mechanism is relatively less efficient than the relaxase-*in cis* mechanism. The well-characterised plasmid family pSK41/pGO1 conjugates to recipients at a frequency of 10^−5^–10^−7^ per donor (10) and relaxase-*in cis* mobilisation by pSK41/pGO1 occurs at similar or slightly lower rates, depending on the particular mobilisable plasmid (52,77). In contrast, while pWBG749 and pBRZ01 transfer at a higher average frequency than pSK41 at 10^−4^–10^−5^ (9,25), the mobilisation rate via the relaxase-*in trans* mechanism is on average only ∼1% of the conjugation frequency of pWBG749. Even a cloned copy of the cognate pWBG749 *oriT* (on pLI749a) facilitates mobilisation at only 2.5% of the rate of pWBG749 (Figure 1C). A strong *cis-*acting preference of a conjugative plasmid's relaxase for its *oriT* has been observed for some rhizobial plasmids (78,79), so this effect is not unique to pWBG749. Given that the relaxase-*in trans* mechanism may be less efficient than the relaxase-*in cis* mechanism, the higher rate of conjugative transfer observed for pWBG749 may raise the relative mobilisation rate to within evolutionarily significant levels. Alternatively, the relaxase-*in trans* mechanism may be more widespread than is currently appreciated, but due to mechanistic preconceptions of mobilisation (17), investigators may have not anticipated the possibility. The relaxase-*in trans* mechanism of mobilisation has been documented for the mobilisation of non-conjugative genomic islands and cryptic chromosomal *oriT* sites by ICEs (27) and plasmids (29,32). Mobilisation of lactococcal plasmids pS7a and pS7b occurs with the help of a helper plasmid pS80, which carries a similar *oriT* sequence and presumably encodes a Mob protein compatible with pS7a and pS7b (30). Additionally a distinct mechanism of mobilisation has been described for *Bacillus subtilis* plasmids, which likely involves interactions between plasmid RCR-Rep proteins and the ICE*Bs*1-encoded T4SS (28). Thus our current appreciation of the variety of mechanisms that facilitate mobilisation of mobile genetic elements is far from comprehensive.

Further investigation into the pWBG749 relaxase-*in trans* mechanism of mobilisation will undoubtedly progress our understanding of the molecular biology, evolution and epidemiology of plasmid transfer in *S. aureus* and other bacteria. The wide prevalence and sequence diversity of pWBG749-like *oriT* sequences and the intramolecular recombination of non-identical *oriT* sequences presents itself as a natural and genetically tractable platform from which to tease apart the roles of *oriT* sequence moieties required for DNA recognition, binding, cleavage, replication and termination of plasmid transfer. From the perspective of molecular evolution and epidemiology, the ability to identify which specific pWBG749-family plasmid mobilises each plasmid based on *oriT-*subfamily type may enable reconstruction of the evolutionary events leading to the emergence of pathogenic strains. In summary, further characterisation of both the mobilisation mechanisms and the epidemiology of pWBG749-family plasmids will undoubtedly expand our understanding of horizontal gene transfer in *S. aureus* and in bacteria in general.

## Supplementary Material

SUPPLEMENTARY DATA
