# Supplementary material for: Origin-of-transfer sequences facilitate mobilisation of non-conjugative antimicrobial-resistance plasmids in Staphylococcus aureus
Source: Nucleic Acids Res. 2015 Aug 3;43(16):7971–83. doi: 10.1093/nar/gkv755 (PMC4652767; doi:10.1093/nar/gkv755)
Supplement: SUPPLEMENTARY DATA [file supp_43_16_7971__index.html]

Origin-of-transfer sequences facilitate mobilisation of non-conjugative antimicrobial-resistance plasmids in Staphylococcus aureus — Origin-of-transfer sequences facilitate mobilisation of non-conjugative antimicrobial-resistance plasmids in Staphylococcus aureus — SUPPLEMENTARY DATA 

# Origin-of-transfer sequences facilitate mobilisation of non-conjugative antimicrobial-resistance plasmids in *Staphylococcus aureus*

## SUPPLEMENTARY DATA

- SUPPLEMENTARY DATA
- SUPPLEMENTARY DATA
- SUPPLEMENTARY DATA
- SUPPLEMENTARY DATA
