# Supplementary material for: Origin-of-transfer sequences facilitate mobilisation of non-conjugative antimicrobial-resistance plasmids in Staphylococcus aureus
Source: Nucleic Acids Res. 2015 Aug 3;43(16):7971–83. doi: 10.1093/nar/gkv755 (PMC4652767; doi:10.1093/nar/gkv755)
Supplement: SUPPLEMENTARY DATA [file supp_gkv755_nar-01460-h-2015-File005_updated.docx]

**Table S1. Bacterial strains and plasmids**

| **Strain/plasmid** | **Description** | **Reference** |
| --- | --- | --- |
| ***Staphylococcus aureus*** |  |  |
| RN4220 | Restriction-deficient derivate of NCTC8325-4 (RN450) | ([1](#_ENREF_1)) |
| WBG4515 | Sm^R^/Nb^R^ derivative of NCTC8325-4 | ([2](#_ENREF_2)) |
| WBG541 | Fus^R^/Rif^R^ derivative of NCTC8325-4 | ([2](#_ENREF_2)) |
| K153N | CC5, ST73 CA-MSSA carrying pWBG747 (Cad^R^, Pen^R^, Bla^+^) | ([3](#_ENREF_3),[4](#_ENREF_4)) |
| WSPP | CC30, ST30-IVc (2B) CA-MRSA carrying pWBG756 (Cad^R^, Pen^R^, Bla^+^) | ([3](#_ENREF_3),[4](#_ENREF_4)) |
| WL6N | CC5, ST5 CA-MSSA carrying pWBG744 (Cad^R^, Pen^R^, Bla^+^) | ([3](#_ENREF_3),[4](#_ENREF_4)) |
| C33S | CC12, ST12-MSSA carrying pWBG761 (Cad^R^, Pen^R^, Bla^+^) | ([3](#_ENREF_3),[4](#_ENREF_4)) |
| K102N | CC45, ST508 CA-MSSA carrying pWBG762 (Cad^R^, Pen^R^, Bla^+^) | ([3](#_ENREF_3),[4](#_ENREF_4)) |
| WBG10526 | WBG541 carrying pWBG749e (pWBG749::Tn*551*) (Em^R^) | ([5](#_ENREF_5)) |
| WB43S | CC5, ST73-IVa (2B) CA-MRSA carrying pWBG745 and pWBG746 (Pen^R^, Em^R^, Cad^R^, Bla^+^) | ([3](#_ENREF_3)) |
| WBG8381 | CC5, ST5-IVa (2B) CA-MRSA containing pWBG749 and pWBG748 (Pen^R^, Bla^+^) | ([3](#_ENREF_3),[4](#_ENREF_4)) |
|  |  |  |
| ***Escherichia coli*** |  |  |
| EPI300 | F^−^ *mcrA* Δ(*mcrCB-hsdSMR-mrr*) (Str^R^) Φ80d*lacZ*ΔM15 Δ*lacX74* *rec*A1 *end*A1 *araD*13*9* Δ(*ara*, *leu*)7697*galU* *galK* λ^−^ *rpsL* *nupG* *trfA* *ton*A *dhfr* | Epicentre |
| **Plasmids** |  |  |
| pLI50 | *S. aureus* (Cm^R^) / *E. coli* (Amp^R^) shuttle vector. | ([6](#_ENREF_6)) |
| pLI749a | pLI50 carrying the *oriT* region of pWBG749 amplified using primers 1 & 2 | This study |
| pLI749b | pLI50 carrying the *oriT* region of pWBG749 amplified using primers 3 & 2 | This study |
| pLI749d | pLI50 carrying the *oriT* region of pWBG749 amplified using primers 4 & 2 | This study |
| pLI749e | pLI50 carrying the *oriT* region of pWBG749 amplified using primers 1 & 5 | This study |
| pLI749f | pLI50 carrying the *oriT* region of pWBG749 amplified using primers 6 & 5 | This study |
| pLI747a | pLI50 carrying the *oriT* region of pWBG747amplified using primers 7 & 8 | This study |
| pLI747b | pLI50 carrying the *oriT* region of pWBG747 amplified using primers 9 & 8 | This study |
| pLI747c | pLI50 carrying the *oriT* region of pWBG747 amplified using primers 10 & 8 | This study |
| pLI747d | pLI50 carrying the *oriT* region of pWBG747 amplified using primers 11 & 8 | This study |
| pLI747e | pLI50 carrying the *oriT* region of pWBG747 amplified using primers 7 & 5 | This study |
| pLI747f | pLI50 carrying the *oriT* region of pWBG747 amplified using primers 6 & 5 | This study |
| pLI744 | pLI50 carrying the OT49-group *oriT* of pWBG744 amplified using primers 12 and 13 | This study |
| pLI747 | pLI50 carrying the OT49-group *oriT* of pWBG747 amplified using primers 12 and 13 | This study |
| pLI756b | pLI50 carrying one of two OT49-group *oriT* sequences of pWBG756 amplified using primers 12 and 13 | This study |
| pLI761a | pLI50 carrying one of two OT49-group *oriT* sequences of pWBG761 amplified using primers 12 and 13 | This study |
| pLI762-49 | pLI50 carrying the OT49-group *oriT* of pWBG762 amplified using primers 12 and 13 | This study |
| pLI762-45 | pLI50 carrying the OT45-group *oriT* of pWBG762 amplified using primers 14 & 15 | This study |
| pLI762-UNa | pLI50 carrying the OTUNa-group *oriT* of pWBG762 amplified using primers 15 & 16 | This study |
| pKY9TOP | pLI50 carrying *oriT-smpO-smpP* region from pWBG749 amplified using primers 1 &17 | This study |
| pKY9TO5P | pKY9TOP with the *smpP* gene replaced with that from pWBG745, sub-cloned from pKY5TOP as a HindII-BamHI fragment. | This study |
| pKY5T | pLI50 carrying *oriT* from pWBG745 amplified using primers 18 &19 | This study |
| pKY5TOP | pLI50 carrying *oriT-smpO-smpP* region from pWBG745amplified using primers 18 &17 | This study |
| pKY5TO9P | pKY5TOP with the *smpP* gene replaced with that from pWBG749, sub-cloned from pKY9TOP as a HindII-BamHI fragment. | This study |
| pKY5TO | pLI50 carrying *oriT-smpO* region from pWBG745amplified using primers 18 & 20 | This study |

**Table S2. Oligonucleotides**

| **Number** | **Name** | **Sequence** |
| --- | --- | --- |
| 1 | 749_5'maximum_EcoRI | ATAAGAATTCATATCAAGCAACAAACAAAATAAGCTA |
| 2 | 749_3'maximum_hindIII | ATATAAGCTTTTCCTAATAAAAATATCTTTTGTTGGCAT |
| 3 | 749_5'_IR3b_EcoRI | ATATGAATTCTAGTGTCACAAAACCGTGACAT |
| 4 | 749_5'NOIR3_ecoRI | ATATGAATTCCTAGGTGTTTTTATGATATCACTATGA |
| 5 | 74bpOriT_rev HindIII | CAGAAGCTTCCGTAAGAGCATAA |
| 6 | 74bpOriT_fwd EcoRI | ATAGAATTCAAAACCCTTGGAATGTCT |
| 7 | 747_5'maximum_EcoRI | ATAAGAATTCAAATTATTATTTAAGACATTAGTGATAACTGA |
| 8 | 747_3'maximum_hindIII | TATAAGCTTTTCTTTAAAACTTCCCCACGTTTTTCT |
| 9 | 747_5'_IR3b_ecoRI | ATATGAATTCTACTGTCTTATTTTTGTGACAAATGCT |
| 10 | 747_5'IR3c_ecoRI | ATATGAATTCTAGTGTCACAAAAGTGTGACA |
| 11 | 5'NOIR3_ecoRI | ATATGAATTCCTACAGCTTTGTATGATATCACTTTAA |
| 12 | Mobilisable_OriT_F | TTATGAATTCGTGACAAATGCTGTATGTA |
| 13 | Mobilisable_OriT_R | TATTAAGCTTGTTTTTCTAAATTCCTGCAG |
| 14 | pWBG762_OT45_5'_EcoRI | ATATGAATTCAAGCAAAATTATTATTTAAGGCA |
| 15 | pWBG762_OT45-OTUNA_3'HindIII | ATATAAGCTTGGCTTTTCTTATCAAAAACTTAGA |
| 16 | pWBG762_OTUNa_5'_EcoRI | ATATGAATTCATCAATCTAGCAATTCACTATCTA |
| 17 | KYE_SmpP3'_BamHI | atatggatccatcatttaactacctttctctatatga |
| 18 | KYE_oriT5'_745_EcoRI | atatGAATTCTAATGTAGAAGAAGCTGAGAAAAAG |
| 19 | KYE_OT45Rev_BamH1 | ATATGGATCCTTCCATAATTCTGTTAACCTCCTAAT |
| 20 | KYE_smpO_BamH1_Rev | ATATGGATCCCATATCACCTACTCATTGATTTAATA |
| 21 | pLI50_seq_F | GAAAAGTGCCACCTGACGTCTAA |
| 22 | pLI50_seq_R | CTATAATCGATAACCACATAACAGTCATA |

**Table S3. Similarities of pWBG749 SmpA-SmpX proteins to those encoded by pMG220, pGL5, pMC189 and pHTβ***

|  | **pWBG749** | | **pMG2200** |  | **pGL5** |  | **pMC189** |  | **pHTβ** |  |  |  |
| --- | --- | --- | --- | --- | --- | --- | --- | --- | --- | --- | --- | --- |
| **Gene name** | **Accession** | **Length (aa)** | **Gene name** | **Identity**  **/coverage** | **Gene name** | **Identity**  **/coverage** | **Gene name** | **Identity**  **/coverage** | **Gene name** | **Identity**  **/coverage** | **Domain similarities/ predicted function /comments** | **Associated references** |
| *smpA* | SAP031A_005 | 101 | pMG2200_103 | 22/60 |  |  | MC28_E014 | 20/66 |  |  | Weak similarity to TraE1 positive regulator of pAD1 transfer | ([7](#_ENREF_7)) |
| *smpB* | SAP031A_004 | 64 |  |  | PGL5p05 | 18/57 | MC28_E011 | 20/50 |  |  |  |  |
| *smpC* | SAP031A_003 | 98 |  |  |  |  |  |  | 80751031 | 17/71 |  |  |
| *smpD* | SAP031A_002 | 359 |  |  |  |  |  |  |  |  |  |  |
| *smpE* | SAP031A_001 | 147 | pMG2200_56 | 41/159 | PGL5p17 | 31/132 |  |  |  |  | Similar to single-stranded DNA binding protein SSB | ([8](#_ENREF_8),[9](#_ENREF_9)) |
| *smpF* | SAP031A_047 | 923 | pMG2200_01 | 94/347 | PGL5p24 | 58/190 | MC28_E004 | 230/571 | 80751034 | 216/620 | Type IV Coupling protein VirD4 | ([10](#_ENREF_10)) |
| *smpG* | SAP031A_046 | 150 |  |  |  |  |  |  |  |  |  |  |
| *smpH* | SAP031A_045 | 613 | pMG2200_02 | 108/418 | PGL5p23 | 107/509 | MC28_E002 | 105/404 | 80751035 | 84/403 | VirB6-like integral membrane protein similar to YddG of ICE*Bs*1 | ([11](#_ENREF_11)) |
| *smpI* | SAP031A_044 | 121 |  |  |  |  |  |  |  |  |  |  |
| *smpJ* | SAP031A_043 | 262 |  |  |  |  | MC28_E185 | 43/171 |  |  |  |  |
| *smpK* | SAP031A_042 | 665 | pMG2200_04 | 101/397 | PGL5p20 | 175/630 | MC28_E181 | 204/489 | 80751039 | 190/658 | Similar to membrane-bound DNA translocation protein VirB4 | ([12](#_ENREF_12)) |
| *smpL* | SAP031A_041 | 368 |  |  |  |  |  |  |  |  | Similar to dual peptidoglycan-hydrolase domain protein TraG of pIP501 | ([13](#_ENREF_13)) |
| *smpM* | SAP031A_040 | 203 |  |  |  |  | MC28_E179 | 38/129 |  |  |  |  |
| *smpN* | SAP031A_039 | 281 |  |  |  |  |  |  | 80751042 | 26/132 | Similar to Orf13 of Tn*916*, contains predicted N-terminal transmembrane domain | ([11](#_ENREF_11)) |
| *smpO* | SAP031A_038 | 84 | pMG2200_104 | 15/50 |  | 0 | MC28_E177 | 13/32 |  |  | CopG/TraN-like Ribbon-Helix-Helix DNA-binding protein | ([14](#_ENREF_14)) |
| *smpP* | SAP031A_037 | 382 | pMG2200_46 | 110/369 | PGL5p12 | 117/358 | MC28_E176 | 140/384 | 80751046 | 117/392 | MOB(MG) relaxase | ([15-18](#_ENREF_15)) |
| *smpQ* | SAP031A_036 | 128 |  |  |  |  | MC28_E175 | 31/117 |  |  |  |  |
| *smpR* | SAP031A_035 | 187 |  |  |  |  |  |  |  |  |  |  |
| *smpS* | SAP031A_034 | 441 | pMG2200_23 | 82/279 |  |  | MC28_E173 | 104/289 | 80751050 | 107/318 | DUF955, conserved HExxH Zinc-binding motif | ([19](#_ENREF_19)) |
| *smpT* | SAP031A_033 | 224 |  |  |  |  |  |  |  |  |  |  |
| *smpU* | SAP031A_032 | 705 | pMG2200_22 | 202/671 |  |  | MC28_E171 | 191/714 | 80751074 | 261/683 | DNA topoisomerase III | ([20](#_ENREF_20)) |
| *smpV* | SAP031A_031 | 449 |  |  |  |  |  |  |  |  |  |  |
| *smpW* | SAP031A_030 | 338 | pMG2200_06 | 93/378 | PGL5p11 | 87/312 | MC28_E170 | 114/328 | 80751054 | 100/322 | DUF3991 and topoisomerase/Primase (Toprim_2) domains | ([21](#_ENREF_21)) |
| *smpX* | SAP031A_029 | 201 |  |  |  |  | MC28_E168 | 71/150 |  |  | Mrr_cat endonuclease domain (COG1787) and DNA topoisomerase I domain (PRK065599) | ([22](#_ENREF_22),[23](#_ENREF_23)) |

*****Accession numbers for plasmid sequences are as follows: pWBG749, GI: 260667549; pMG2200, GI: 217388333; pGL5, GI: 380236490; pMC189, GI: 407702348; pHTβ, GI: 80751013.


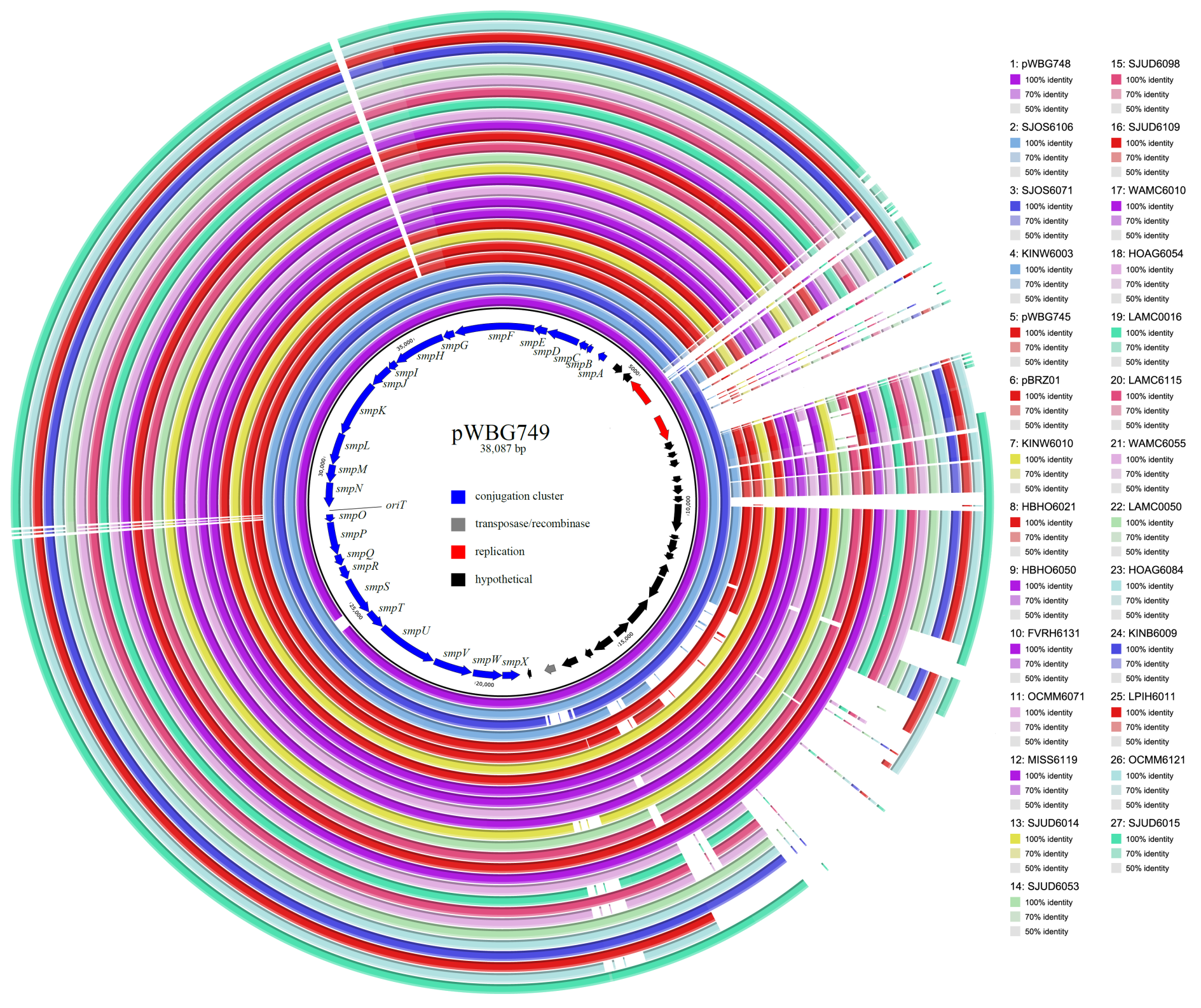
**Figure S1. Nucleotide comparison of pWBG749-like conjugative plasmids.**

pWBG749 was used as a reference sequence in a BLASTN alignment ([24](#_ENREF_24))
(-ungapped mode) displayed using BRIG ([25](#_ENREF_25)). Plasmids pWBG748, pWBG745, and pBRZ01 (GI:260066070, GI:260667549 and Bioproject ID 183704) were used as query sequences along with contigs carrying *smpA-smpX* from the MRSA Orange County initiative of the Broad Institute (broadinstitute.org). Genes (shown as arrows) are coloured according to predicted functions outlined in the inset key. The legend on the right indicates colours used for each strain for each ring, numbered from innermost to outermost ring.


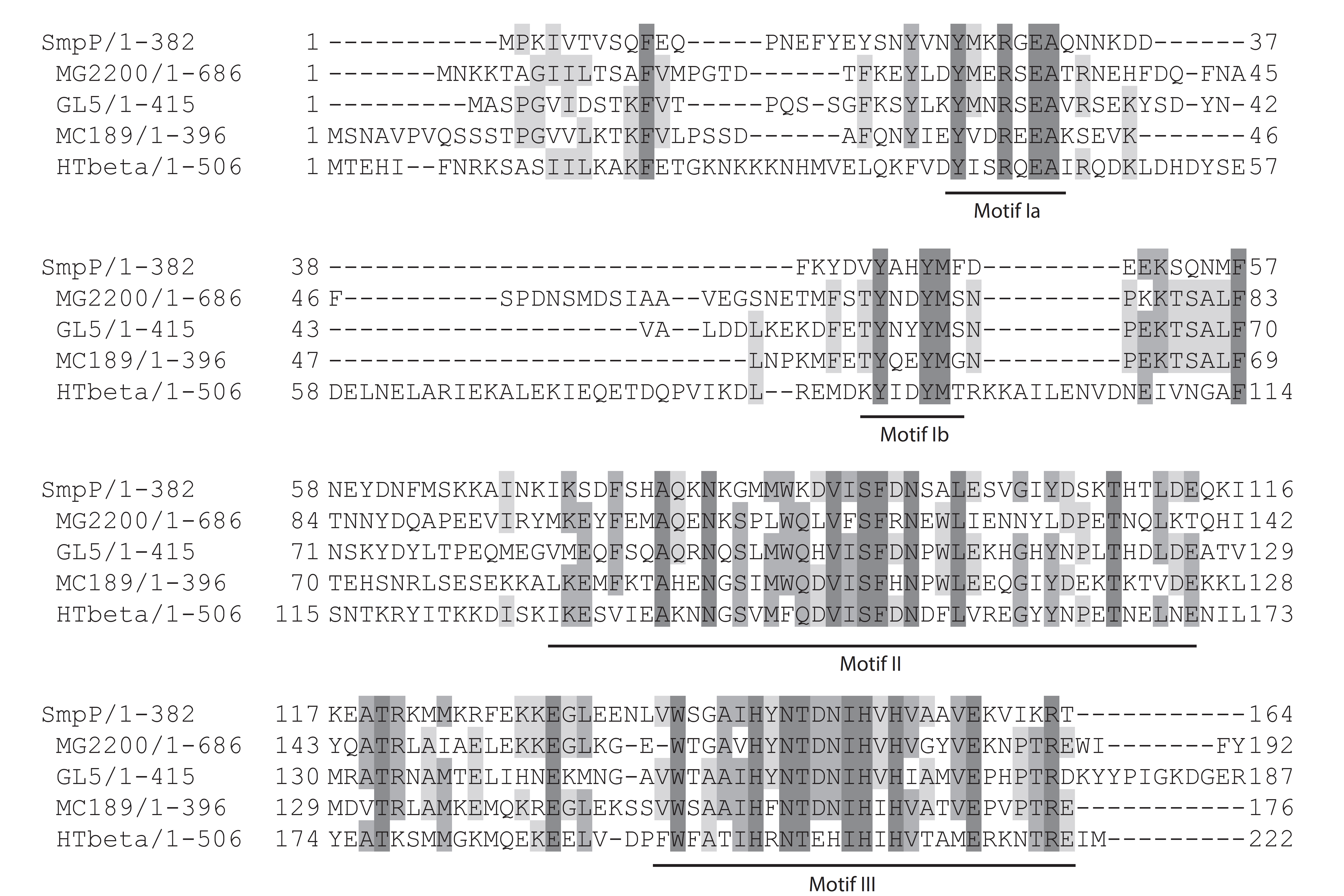


**Figure S2. Conserved MOB_MG_-family motifs in the SmpP sequence.**

T-coffee (mcoffee mode) alignment of the N-terminal regions of the putative relaxase proteins encoded by conjugative plasmids pWBG749 (SmpP), pHTβ, pGL5, pMC189 and pMG2200. Underlined are motifs conserved within the MOB_MG_ family of relaxases ([16](#_ENREF_16)), including two tyrosine containing motifs (motifs Ia and Ib) and the histidine triad motif (Motif III) W[x4]H[x2]T[x3]HUH[x4]E[x4]R. Conserved residues are indicated by shading (darker shading indicates higher conservation).


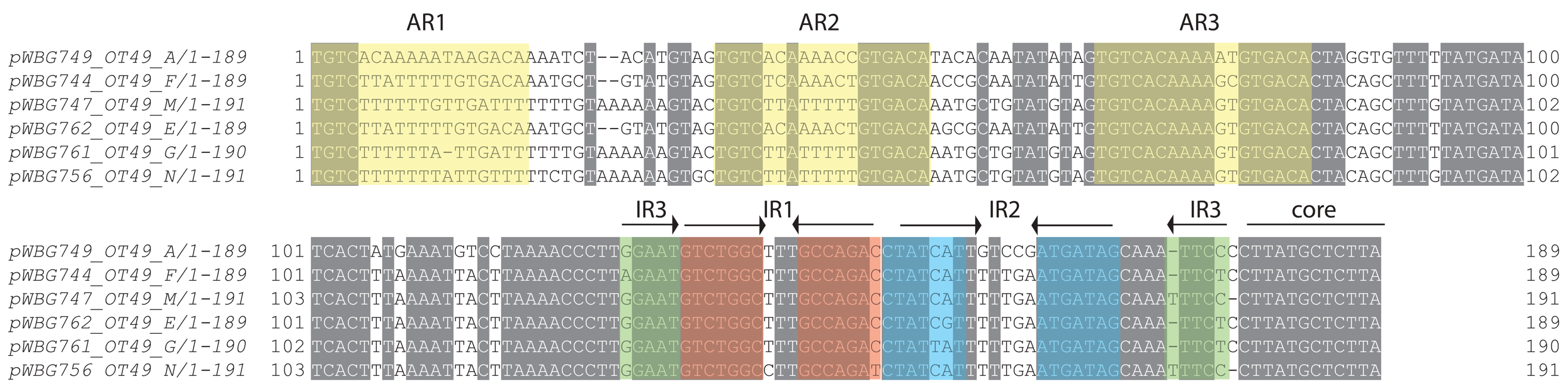


**Figure S3. Alignment of pWBG749-like *oriT* sequences identified on plasmids mobilisable by pWBG749e**

A DNA alignment (using T-coffee in rcoffee mode ([26](#_ENREF_26),[27](#_ENREF_27))) of the pWBG749 *oriT* with *oriT* regions identified on pWBG762, pWBG744, pWBG747, pWBG761 and pWBG756. The same regions were cloned into pLI50 and used in mobilisation experiments with pWBG749 (Table 1).


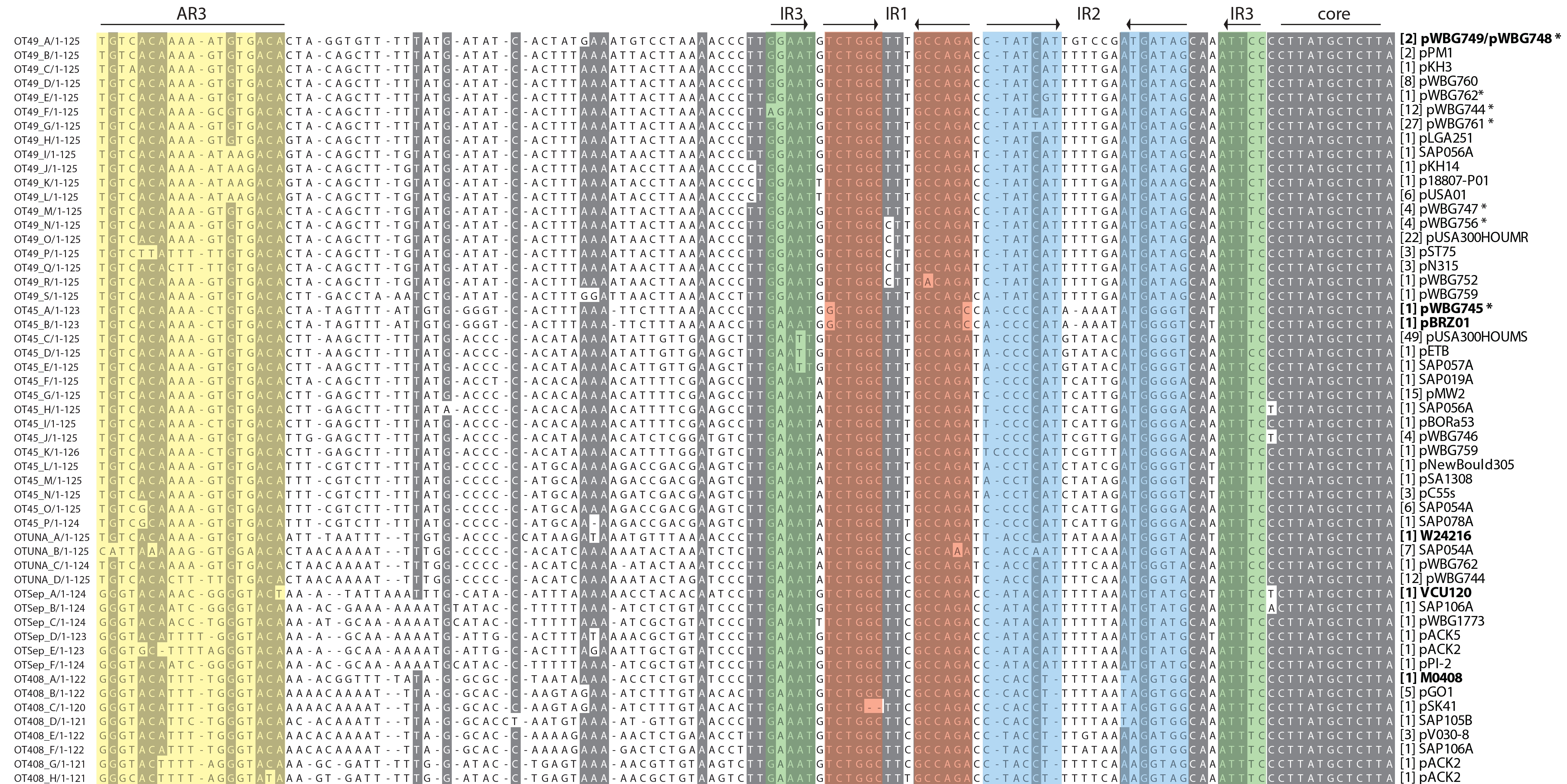


**Figure S4. Multiple sequence alignment of 53 distinct *oriT* sequences identified on conjugative and non-conjugative plasmids.** Two-hundred and twenty-nine identified *oriT* regions were aligned from the AR3 motif to the core sequence (using T-coffee in rcoffee mode ([26](#_ENREF_26),[27](#_ENREF_27))), resulting in the identification of 53 unique sequences. Shaded regions (grey background with white text) indicate positions with >90% conservation. Colour shading indicates AR, IR1, IR2 and IR3 motifs as coloured in other figures. Tree construction (Fig. S5) and alignment comparisons revealed that the *oriT* sequences contained distinct IR2 sequences that corresponded to the *oriT* sequences found on 5 pWBG749-family conjugation clusters (pWBG749-family plasmid names/strains are in bold on the right, numbers in square brackets indicate the number of sequences identified with the particular variant). The 5 *oriT* groups were named OT49 (pWBG749), OT45 (pWBG745), OTUNa (*S. aureus* W24216), OTSep (*S. epidermidus* VCU120) and OT408 (*S. aureus* M0408). Each ‘OT’ group is named on the left and unique variants are named alphabetically for each OT group. A full list of *oriT* sequences, their OT groupings and accession numbers can be found in Supplementary dataset S2. *****Asterisks indicate that the sequence was cloned and successfully mobilised by pWBG749e in this work (Note that mobilisation of OT45_A required the presence of pWBG745 *smpO*).





**Figure S5. Maximum likelihood tree of *oriT* sequences**

The DNA sequence alignment from Fig. S4 was used for tree construction using PhyML software ([28](#_ENREF_28)) with default settings. The tree was constructed using a neighbour-joing starting tree with no outgroup. Bootstrap support for major nodes is shown (1,000 trees generated). The same major nodes were produced when a maximum-parisimony starting tree was used. *oriT* sequences belonging to conjugative plasmids or on predicted conjugation-gene clusters are shown in bould.


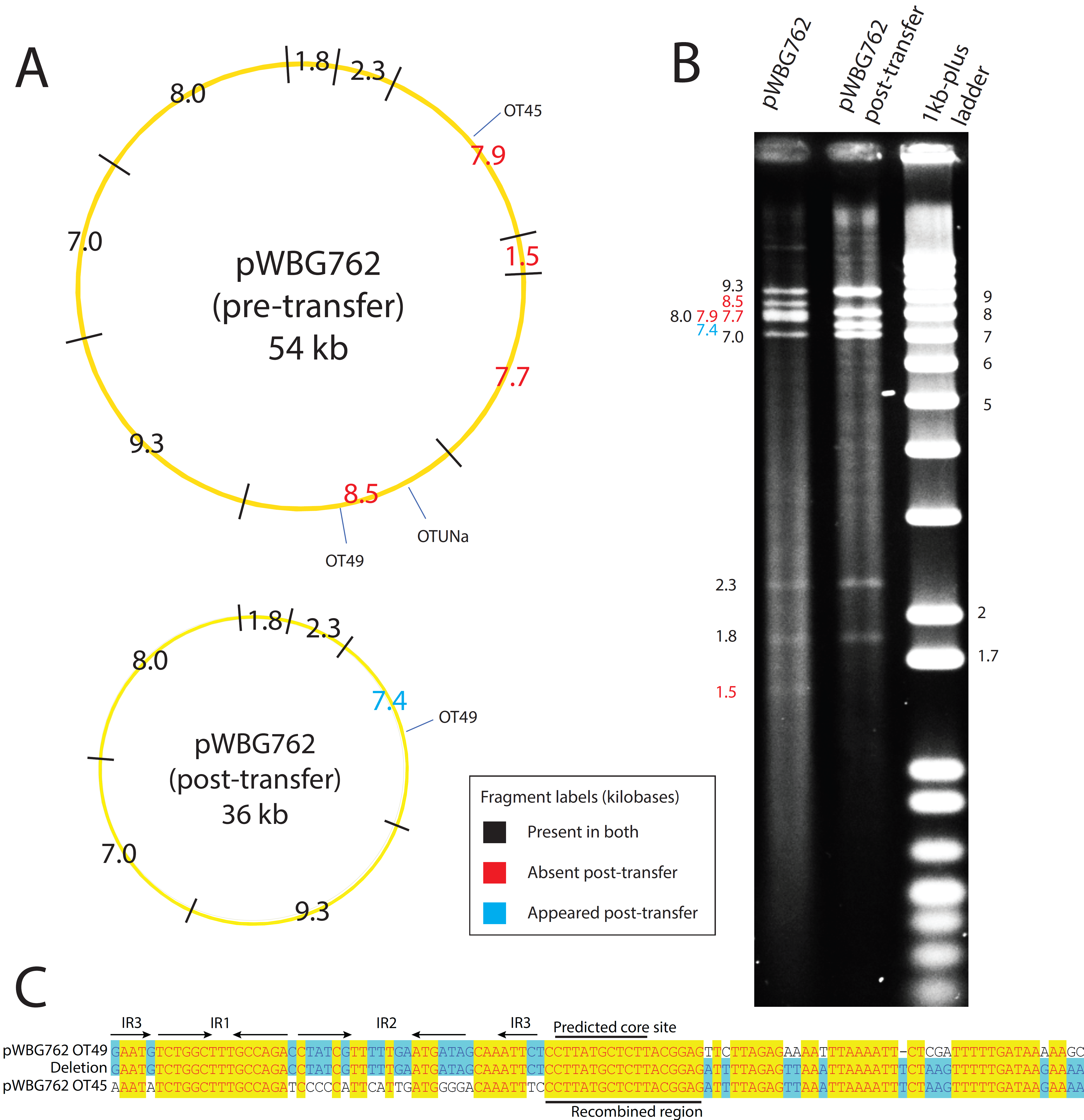


**Figure S6. pWBG749e-mediated deletion between OT49 and OT45 *oriT* sites.**

(*A*) Plasmid diagrams of pWBG762 before and after conjugative mobilisation by pWBG749e. Black marks on plasmid maps indicate EcoRI sites. Positions of *oriT* sites are labelled by their ‘OT’ group. Sizes of fragments are indicated in black text, or in red text if the fragments were lost following mobilisation, or in blue text for the 7.4-kb EcoRI fragment that evolved following pWBG749e-mediated deletion. (*B*) Agarose gel DNA electrophoresis of EcoRI-digested pWBG762 DNA before and after conjugative mobilisation by pWBG749. Lanes are labelled above each lane. All sizes are in kilobases and are coloured as in part A. (*C*) Alignment of the OT49-type and OT45-type *oriT* sequences of pWBG762 and the sequence of the PCR product spanning the deletion that occurred following mobilisation by pWBG749e.

**References**

1. Kreiswirth, B.N., Lofdahl, S., Betley, M.J., O'Reilly, M., Schlievert, P.M., Bergdoll, M.S. and Novick, R.P. (1983) The toxic shock syndrome exotoxin structural gene is not detectably transmitted by a prophage. *Nature*, **305**, 709-712.

2. Townsend, D.E., Grubb, W.B. and Ashdown, N. (1983) Gentamicin resistance in methicillin-resistant *Staphylococcus aureus*. *Pathology*, **15**, 169-174.

3. O'Brien, F.G., Coombs, G.W., Pearman, J.W., Gracey, M., Moss, F., Christiansen, K.J. and Grubb, W.B. (2009) Population dynamics of methicillin-susceptible and -resistant *Staphylococcus aureus* in remote communities. *J Antimicrob Chemother*, **64**, 684-693.

4. Shearer, J.E., Wireman, J., Hostetler, J., Forberger, H., Borman, J., Gill, J., Sanchez, S., Mankin, A., Lamarre, J., Lindsay, J.A. *et al.* (2011) Major families of multiresistant plasmids from geographically and epidemiologically diverse staphylococci. *G3*, **1**, 581-591.

5. O'Brien, F.G., Ramsay, J.P., Monecke, S., Coombs, G.W., Robinson, O.J., Htet, Z., Alshaikh, F.A.M. and Grubb, W.B. (2015) *Staphylococcus aureus* plasmids without mobilization genes are mobilized by a novel conjugative plasmid from community isolates. *J Antimicrob Chemother*, **70**, 649-652.

6. Lee, C.Y., Buranen, S.L. and Ye, Z.H. (1991) Construction of single-copy integration vectors for *Staphylococcus aureus*. *Gene*, **103**, 101-105.

7. Pontius, L.T. and Clewell, D.B. (1992) Conjugative transfer of *Enterococcus faecalis* plasmid pAD1: nucleotide sequence and transcriptional fusion analysis of a region involved in positive regulation. *J Bacteriol*, **174**, 3152-3160.

8. Sundberg, C.D. and Ream, W. (1999) The *Agrobacterium tumefaciens* chaperone-like protein, VirE1, interacts with VirE2 at domains required for single-stranded DNA binding and cooperative interaction. *J Bacteriol*, **181**, 6850-6855.

9. Raghunathan, S., Kozlov, A.G., Lohman, T.M. and Waksman, G. (2000) Structure of the DNA binding domain of *E. coli* SSB bound to ssDNA. *Nat Struct Biol*, **7**, 648-652.

10. Hamilton, C.M., Lee, H., Li, P.L., Cook, D.M., Piper, K.R., von Bodman, S.B., Lanka, E., Ream, W. and Farrand, S.K. (2000) TraG from RP4 and TraG and VirD4 from Ti plasmids confer relaxosome specificity to the conjugal transfer system of pTiC58. *J Bacteriol*, **182**, 1541-1548.

11. Alvarez-Martinez, C.E. and Christie, P.J. (2009) Biological diversity of prokaryotic type IV secretion systems. *Microbiol Mol Biol Rev*, **73**, 775-808.

12. Thompson, D.V., Melchers, L.S., Idler, K.B., Schilperoort, R.A. and Hooykaas, P.J. (1988) Analysis of the complete nucleotide sequence of the *Agrobacterium tumefaciens virB* operon. *Nucleic Acids Res*, **16**, 4621-4636.

13. Arends, K., Celik, E.K., Probst, I., Goessweiner-Mohr, N., Fercher, C., Grumet, L., Soellue, C., Abajy, M.Y., Sakinc, T., Broszat, M. *et al.* (2013) TraG encoded by the pIP501 type IV secretion system is a two-domain peptidoglycan-degrading enzyme essential for conjugative transfer. *J Bacteriol*, **195**, 4436-4444.

14. Hernandez-Arriaga, A.M., Rubio-Lepe, T.S., Espinosa, M. and del Solar, G. (2009) Repressor CopG prevents access of RNA polymerase to promoter and actively dissociates open complexes. *Nucleic Acids Res*, **37**, 4799-4811.

15. Aguado-Urda, M., Gibello, A., Blanco, M.M., Lopez-Campos, G.H., Cutuli, M.T. and Fernandez-Garayzabal, J.F. (2012) Characterization of plasmids in a human clinical strain of *Lactococcus garvieae*. *PLoS One*, **7**, e40119.

16. Zheng, B., Tomita, H., Inoue, T. and Ike, Y. (2009) Isolation of VanB-type *Enterococcus faecalis* strains from nosocomial infections: first report of the isolation and identification of the pheromone-responsive plasmids pMG2200, Encoding VanB-type vancomycin resistance and a Bac41-type bacteriocin, and pMG2201, encoding erythromycin resistance and cytolysin (Hly/Bac). *Antimicrob Agents Chemother*, **53**, 735-747.

17. Guan, P., Ai, P., Dai, X., Zhang, J., Xu, L., Zhu, J., Li, Q., Deng, Q., Li, S., Wang, S. *et al.* (2012) Complete genome sequence of *Bacillus thuringiensis* serovar Sichuansis strain MC28. *J Bacteriol*, **194**, 6975.

18. Tomita, H. and Ike, Y. (2005) Genetic analysis of transfer-related regions of the vancomycin resistance *Enterococcus* conjugative plasmid pHTbeta: identification of *oriT* and a putative relaxase gene. *J Bacteriol*, **187**, 7727-7737.

19. Jongeneel, C.V., Bouvier, J. and Bairoch, A. (1989) A unique signature identifies a family of zinc-dependent metallopeptidases. *FEBS Lett*, **242**, 211-214.

20. Zhang, H.L., Malpure, S. and DiGate, R.J. (1995) *Escherichia coli* DNA topoisomerase III is a site-specific DNA binding protein that binds asymmetrically to its cleavage site. *J Biol Chem*, **270**, 23700-23705.

21. Aravind, L., Leipe, D.D. and Koonin, E.V. (1998) Toprim--a conserved catalytic domain in type IA and II topoisomerases, DnaG-type primases, OLD family nucleases and RecR proteins. *Nucleic Acids Res*, **26**, 4205-4213.

22. Waite-Rees, P.A., Keating, C.J., Moran, L.S., Slatko, B.E., Hornstra, L.J. and Benner, J.S. (1991) Characterization and expression of the *Escherichia coli* Mrr restriction system. *J Bacteriol*, **173**, 5207-5219.

23. Tse-Dinh, Y.C. and Beran-Steed, R.K. (1988) *Escherichia coli* DNA topoisomerase I is a zinc metalloprotein with three repetitive zinc-binding domains. *J Biol Chem*, **263**, 15857-15859.

24. Camacho, C., Coulouris, G., Avagyan, V., Ma, N., Papadopoulos, J., Bealer, K. and Madden, T.L. (2009) BLAST+: architecture and applications. *BMC Bioinformatics*, **10**, 421.

25. Alikhan, N.F., Petty, N.K., Ben Zakour, N.L. and Beatson, S.A. (2011) BLAST Ring Image Generator (BRIG): simple prokaryote genome comparisons. *BMC Genomics*, **12**, 402.

26. Notredame, C., Higgins, D.G. and Heringa, J. (2000) T-Coffee: A novel method for fast and accurate multiple sequence alignment. *J Mol Biol*, **302**, 205-217.

27. Wilm, A., Higgins, D.G. and Notredame, C. (2008) R-Coffee: a method for multiple alignment of non-coding RNA. *Nucleic Acids Res*, **36**, e52.

28. Guindon, S. and Gascuel, O. (2003) A simple, fast, and accurate algorithm to estimate large phylogenies by maximum likelihood. *Syst Biol*, **52**, 696-704.
